# Female authors in top-rank journals of different medical specialties

**DOI:** 10.1186/cc9957

**Published:** 2011-03-11

**Authors:** K Amrein, C Putz-Bankuti, J Mader, A Amegah-Sakotnik, T Urbanic, D Wagner, E Prandl, N Sareban, S Amrein, A Langmann

**Affiliations:** 1 Medical University of Graz, Austria

## Introduction

In various scientific fields, including medical research, men have been found to have a higher scientific output than women. These differences may be due to women's lower integration in the scientific community [1]. Even though the proportion of female authors has increased in the past decades, women still contribute less to prominent medical journals [2].

## Methods

Thirty-five top-10-ranked journals of eight different medical categories were analysed: Medicine, General & Internal (M,GI), Critical Care (CC), Anaesthesiology (A), Surgery (S), Emergency Medicine (EM), Radiology (R), Haematology (H) and Clinical Neurology (N). Over a 12-month period, we evaluated the first and senior authors' first name for gender.

## Results

Thirty-one percent of evaluable first authors were female, compared with 18% of all senior authors. There were significant differences between the evaluated categories, with the lowest percentage of female first authors in the category Surgery, followed by Emergency Medicine (Table 1). In every category, the proportion of female senior authors was significantly lower than that of first authors.

**Table 1:** Percentage of female authors

	First authors	Senior authors
S	20.8	12.8
EM	22.1	15.3
CC	25.6	13.8
M,GI	30.3	19.8
R	35.1	17.1
H	35.7	17.2
N	38.6	17.1
A	38.7	23.6

## Conclusions

There is a wide variation in the proportion of contributing female authors between the subspecialties analysed, probably reflecting the varying percentage of female scientists. However, in all evaluated medical categories, the proportion of papers authored by females was significantly lower than those authored by men.
